# Clinical features and biomarkers of semantic variant primary progressive aphasia with MAPT mutation

**DOI:** 10.1186/s13195-023-01176-y

**Published:** 2023-01-27

**Authors:** Jing Xu, Yanmin Xia, Meng Meng, Fang Liu, Ping Che, Yanxin Zhang, Ying Wang, Li Cai, Wen Qin, Nan Zhang

**Affiliations:** 1 Department of Neurology, Tianjin Neurological Institute, Tianjin Medical University General Hospital, 154 Anshan Road, Heing District, Tianjin, 300052 China; 2 Department of Neurology, Affiliated Hospital of Hebei University, Baoding, 071000 Hebei China; 3 Department of Neurology, Tianjin Medical University General Hospital Airport Site, Tianjin, China; 4 Department of PET-CT Diagnostic, Tianjin Medical University General Hospital, Tianjin, 300052 China; 5 Department of Radiology and Tianjin Key Laboratory of Functional Imaging, Tianjin Medical University General Hospital, Tianjin, China

**Keywords:** Frontotemporal lobar degeneration, Semantic variant primary progressive aphasia, MAPT gene, P301L mutation, Next-generation sequencing

## Abstract

**Background:**

Semantic variant primary progressive aphasia (svPPA) is generally sporadic, with very few reports of tau pathology caused by MAPT mutations.

**Methods:**

A 64-year-old man was diagnosed with svPPA with MAPT P301L mutation. Clinical information, cognitive and language functions, multimodal magnetic resonance imaging (MRI), blood biomarkers, fluorodeoxyglucose (FDG) imaging and tau positron emission tomography (PET) were obtained.

**Results:**

Semantic memory impairment was the earliest and most prominent symptom in this family. Tau accumulation and hypometabolism were observed prior to brain atrophy in mutation carriers. Plasma NfL and GFAP concentrations were elevated in the two svPPA patients. Some relative decreases and some relative increases in regional cerebral blood flow (CBF) as measured by arterial spin labelling (ASL) were observed in mutation carriers compared to noncarriers.

**Conclusions:**

This study describes a large svPPA-affected family with the MAPT P301L mutation and provides an ideal model for inferring underlying pathology and pathophysiological processes in svPPA caused by tauopathies.

## Background

Frontotemporal lobar degeneration (FTLD) is second only to Alzheimer’s disease (AD) as the most common cause of early-onset dementia [1, 2]. According to its clinical features, FTLD is usually classified into two major phenotypes: behavioural variant frontotemporal dementia (bvFTD) [3] and primary progressive aphasia (PPA) [4]. The latter includes nonfluent/agrammatic variant (nfvPPA), semantic variant (svPPA) and logopenic variant PPA (lvPPA, of which most cases are linked to AD pathology). Pathologically, nearly all FTLD patients exhibit positive immunostaining for 1 of 3 major protein groups: tau (FTLD-Tau, 40–45%), TAR DNA-binding protein 43 (FTLD-TDP, 40–45%) or fused in sarcoma (FTLD-FUS, 5%) [5]. However, the relationships among pathologies and clinical phenotypes are quite complex.

Among FTLD spectrum disorders, svPPA is the most consistently defined phenotype associated with loss of knowledge regarding words and objects and difficulty in naming and is usually caused by FTLD-TDP pathology [6, 7]. Moreover, although a family history of dementia has been reported in up to 40% of FTLD patients [8], svPPA is typically sporadic with the least heritability compared with nfvPPA and bvFTD [9].

Several mutations across three genes, including chromosome 9 open reading frame 72 (C9orf72), progranulin (GRN) and microtubule-associated protein tau (MAPT), account for the majority of inherited FTLD cases [10]. MAPT mutations are the only known genetic aetiologies of FTLD-Tau, which may cause variable cognitive (executive and verbal), behavioural and motor (e.g. parkinsonism) deficits [11–14]. In 1998, mutations in the MAPT gene were first described as a cause of dementia linked to tau pathology, which was named frontotemporal dementia and parkinsonism linked to chromosome 17 (FTDP-17) [15]. To date, more than 60 different pathogenic missense, silent and intronic MAPT mutations have been identified [16].

P301L is the first identified mutation in the MAPT gene with clinical and pathologic heterogeneity. According to previous publications, bvFTD is the most common clinical presentation attributed to the MAPT P301L mutation worldwide [17–20]. To date, only one publication has reported a family with familial svPPA caused by MAPT mutation [21]. Here, we comprehensively describe the clinical, neuropsychological, language, structural magnetic resonance imaging (MRI) and ^18^F-labelled fluorodeoxyglucose (FDG) and tau positron emission tomography (PET) features of svPPA patients and presymptomatic carriers in a Chinese family with a confirmed MAPT P301L mutation. We further assessed the differences in plasma biomarkers and cerebral blood flow (CBF) measured with arterial spin labelling (ASL) MRI between mutation carriers and noncarriers to explore the early biological changes in the svPPA phenotype that are caused by MAPT mutation.

## Methods

### Proband (patient III-12)

Patient III-12 is the proband in the present study. The patient is a 64-year-old male Chinese farmer with 9 years of education and no history of neurological disease or psychiatric disorders, such as depression, schizophrenia or substance abuse. At 62 years of age, he gradually began to exhibit slow reactions, a decline in the ability to name common objects and memory loss. Two years later, he became unable to understand the meanings of many words and made unsuitable word choices. When visiting our memory clinic, he displayed a polite attitude and had a respectable and proper appearance. He was unable to follow simple instructions and preserved only a few stereotypically verbal expressions and basic speech comprehension. He had no pyramidal or cerebellar signs at that time. MRI images obtained at another hospital revealed atrophy in the bilateral medial and lateral temporal lobes. A clinical diagnosis of svPPA was presumed according to the diagnostic criteria published in 2011 [4]. Since several family members across four generations exhibited similar symptoms, next-generation sequencing (NGS) and repeat-primed PCR were performed on this patient. The results indicated that the proband carried the exon 10 MAPT P301L variant but not C9orf72 expansions.

### Participants

The study was approved by the Ethics Committee of the Tianjin Medical University General Hospital. Informed consent for the study was obtained from all participants. We reviewed the detailed demographic and clinical information of all subjects in the pedigree and summarized the clinical features of both the living and dead subjects with suspected svPPA. Apart from the proband, 17 other family members (III-13, IV-2, IV-8, IV-10, IV-11, IV-30, IV-31, IV-32, IV-6, IV-14, IV-15, IV-22, IV-27, IV-33, IV-34, IV-37, and IV-38) from two generations of the family consented to participate in this study. Detailed clinical history collections, neurological examinations, cognitive and language assessments, blood samples for cosegregation analysis and biomarker assays, and multimodal MRI scans were performed. In addition, PET scans were obtained from subjects IV-2, IV-22 and IV-30. All participants in this study were of Han Chinese ethnicity.

### Genetic analyses

Genomic DNA was extracted from peripheral blood leukocytes by using standard methods. NGS was performed on the proband (III-12) using the Illumina HiSeq2000 platform (Beijing Genomics Institute, Beijing, China) and verified by Sanger sequencing. C9orf72 genotyping was accomplished using repeat-primed PCR and sequencing, which allows the detection of approximately 30 repeats [22]. A cosegregation analysis was performed for all participants.

### Neuropsychological and language assessments

Neuropsychological testing was conducted within 2 weeks prior to neuroimaging by a trained investigator who was blinded to the clinical conditions and genetic analyses. All participants underwent an assessment battery for cognitive and language functions, activities of daily living and behavioural and psychological symptoms as previously described [23, 24], which included the Mini-Mental State Examination (MMSE), Montreal Cognitive Assessment (MoCA), Auditory Verbal Learning Test (AVLT), Brief Visuospatial Memory Test–Revised (BVMT-R), Digit Span Test, Symbol Digit Modalities Test (SDMT), Trail Making Test-A (TMT-A) and TMT-B, Stroop Colour–Word Interference Test [25], Controlled Oral Word Association Test (COWAT), Animal Fluency Test (AFT), Boston Naming Test (BNT), 20-item Activities of Daily Living Scale (ADL), Neuropsychiatric Inventory (NPI) and an additional comprehensive language evaluation using the Aphasia Battery of Chinese (ABC) [26].

### MRI acquisition

Brain MRI was obtained for all participants except for the proband and subject IV-27 because of poor cooperation or claustrophobia. Images were collected using a 3.0-Tesla MRI scanner (Discovery MR750, General Electric, Milwaukee, WI, USA) with a 64-channel phased array head coil. We first performed a coronal T1-weighted 3D brain volume (BRAVO) sequence to serve as a template for coregistration with the ASL imaging data: echo time/repetition time (TE/TR): 3.2 ms/8.2 ms, flip angle (FA): 12°, field of view (FOV): 256×256 mm^2^, matrix size: 256×256, NEX=1, slice thickness: 1.0 mm and number of slices: 188. Whole-brain perfusion was measured using the 3D pseudocontinuous ASL series: TE/TR: 11.1 ms/5046 ms, labelling duration: 1450 ms, post-labelling delay: 2025 ms, FA: 155°, matrix size: 128×128, FOV: 240×240 mm^2^, arms=8, acquisition points=512, slice thickness: 3 mm and number of slices: 50. The subjects took no drugs that might affect CBF regulation within the previous 2 weeks and abstained from alcohol, caffeine and nicotine for at least 6 h before the MRI. T1-weighted, T2-weighted and fluid-attenuated inversion recovery (FLAIR) images were used to evaluate brain atrophy and lesions.

### PET image acquisition

PET scans were performed with a GE Discovery LS PET/CT scanner in a 3D scanning mode in the PET/CT centre of the Tianjin Medical University General Hospital. Cerebral glucose metabolism and tau distribution were measured by ^18^F-FDG and ^18^F-S16 PET, respectively, for subjects IV-2, IV-22 and IV-30 at 1-week intervals according to a previously described procedure [27].

^18^F-S16, which is a novel tau radiotracer, has been demonstrated to have high affinity and selectivity for tangles over amyloid-β (Aβ) in preclinical studies [28], with prominent uptake in the cortex, where abnormal tau aggregates are expected to accumulate in patients with AD, although off-target binding has also been observed in the basal ganglia and brain stem per a previous report [27]. In the present study, ^18^F-S16 was injected into an antecubital vein at a dose of 383.6 ± 30.6 MBq. After 70 min, PET scans were acquired with an acquisition scheme that consisted of 5 frames (6×300 s). Five days later, the subjects fasted for at least 6 h, received intravenous injections of 185–259 MBq ^18^F-FDG and waited in a darkened, quiet room. At 40 min after the ^18^F-FDG injections, PET emission scans were performed. Each frame produced 35 slices of 4.25-mm thickness, which covered the entire brain. The images were reconstructed to a 128×128 matrix (pixel size of 2.5×2.5 mm^2^).

### Visual rating of PET images

The PET images were visually read by two experienced nuclear medicine physicians (Li Cai and Ying Wang) who were blinded to the clinical data. Visual ratings of the ^18^F-FDG and ^18^F-S16 PET scans were conducted according to a previously described procedure [27, 29]. In brief, the FDG frames were normalized to the mean activity in the pons and were then presented using the NIH colour scale; they could be windowed and viewed in 3 planes at the raters’ discretion. In terms of S16, the individual grey matter (GM) regions of interest were applied to the dynamic images in the native PET space through an intermediate MRI-to-PET coregistration step using SPM12 to preserve the high resolution of the PET data. The cerebellar cortex was used as the reference region to quantify the binding of the S16 tracer.

### Measurement of plasma markers

Plasma levels of neurofilament light chain (NfL) and glial fibrillary acidic protein (GFAP) were measured using single molecular array technology with an ultrahigh sensitivity protein molecular detection instrument (Simoa HD-1, Quanterix, MA, USA) with a Neurology 2-Plex B kit (502713, Quanterix, MA, USA) according to a previously described procedure [30].

### ASL image processing

Image analyses were performed using statistical parametric mapping (SPM12, Institute of Neurology, London, UK) software and MATLAB (Version R2015a; MathWorks, Natick, MA, USA) on a Windows computer according to a previously described procedure [31]. All images underwent manual quality control by a trained researcher for image quality and successful coregistration. To identify the changes in global and regional CBF, ASL images were automatically converted into CBF maps with FuncTool software (version 9.4, GE Medical Systems) on an Advantage Windows workstation. Image preprocessing was conducted as follows: (1) the CBF images were registered to the structural MRI images; (2) the structural MRI images were normalized to the standard Montreal Neurological Institute (MNI) brain template and segmented into probability maps of white matter (WM), GM and cerebrospinal fluid (CSF); (3) normalized CBF images were constructed using the parameters determined from the structural images and multiplied by a binary brain tissue mask consisting only of GM and WM; and (4) to decrease the interindividual anatomical differences, the normalized CBF images were then smoothed by using a Gaussian kernel with a full width at half maximum (FWHM) of 10 mm.

Voxel-based analysis was performed with a GM mask using SPM12 software according to a previously described procedure [31]. The CBF differences between mutation carriers and noncarriers were compared by using a two-sample *t* test model, which used age and sex as covariates. The coordinates are reported in the standard MNI anatomical space. With a voxel-level peak threshold of *P* < 0.001 (uncorrected) over whole brain regions, we primarily identified clusters >100 voxels (voxel size =1.5 mm×1.5 mm×1.5 mm) for the analysis of both absolute CBF and after adjusting for global values with ANCOVA. Significant regions were localized with Talairach–Daemon software (Research Imaging Center, University of Texas Health Science Center, San Antonio, TX, USA). The nearest GM locations are reported for all of these regions. The SPM maps for CBF were overlaid on a standard T1-weighted MRI brain template in stereotaxic space. To quantify the CBF changes in specific cortical regions, we used a 4-mm radius spherical volume of interest (VOI) centred at the peak voxel of those clusters that were significant in the SPM analyses. We then obtained the relative CBF values by calculating the ratios of the VOI values to the global CBF values in all participants with SPM12.

### Statistical analysis

For cross-sectional analyses, we identified two groups: 9 mutation carriers (including both symptomatic and presymptomatic subjects) and 9 mutation noncarriers. NfL levels were log transformed for further analysis because they were not normally distributed. The differences in the plasma biomarker levels between the two groups were evaluated using general linear models, which were adjusted for age because both NfL and GFAP expression levels were correlated with age. Spearman’s correlation coefficients were used to analyse the relationship between plasma NfL and GFAP. The global CBF and relative values of regional CBF were compared between the two groups by using a two-sample *t* test. Statistical significance was set at the *P*<0.05 level. All of the statistical analyses were performed using SPSS 19.0 software (SPSS Inc., Chicago, IL).

## Results

### Family history, clinical manifestations and genetic testing

The dementia histories were retrospectively reviewed for all available family members, including both living and deceased subjects, by drawing from previous medical records and supplementary information from caregivers. III-5 (deceased, female) exhibited difficulty in naming and had a bad temper and excessive appetite since 48 years of age. She was unable to take care of herself 4 years after the disease onset and died at 55 years of age. III-1 (deceased, male) was 59 years old at the time of onset and exhibited difficulty with naming in the early stage. As the disease progressed, he could not understand familiar words, such as “aunt.” He died at 65 years of age. III-9 (deceased, female) presented with gradually worsening difficulty in word finding and naming from 55 years of age and died at the age of 62. Family members recalled that I-2 (female), II-3 (age of onset 60 years, male) and III-11 (age of onset 52 years, male) also had dementia (although no detailed clinical information was available) and that they died from unknown causes. No deceased subjects with dementia showed obvious executive dysfunction, at least in the early stages, although there was no objective neuropsychological evidence regarding these persons.

IV-22 (50 years old, female) developed a progressive language disability with gradual onset over the course of 1 year. She forgot the names of familiar friends and relatives and became incapable of understanding the meaning of simple words, such as “computer” or “skyscraper,” and common Chinese idioms but had no psychiatric or behavioural changes. Motor deficits and apraxia were absent. This subject was diagnosed with svPPA according to her symptoms and brain MRI and was confirmed by genetic testing to carry the MAPT P301L mutation.

**Fig. 1:**
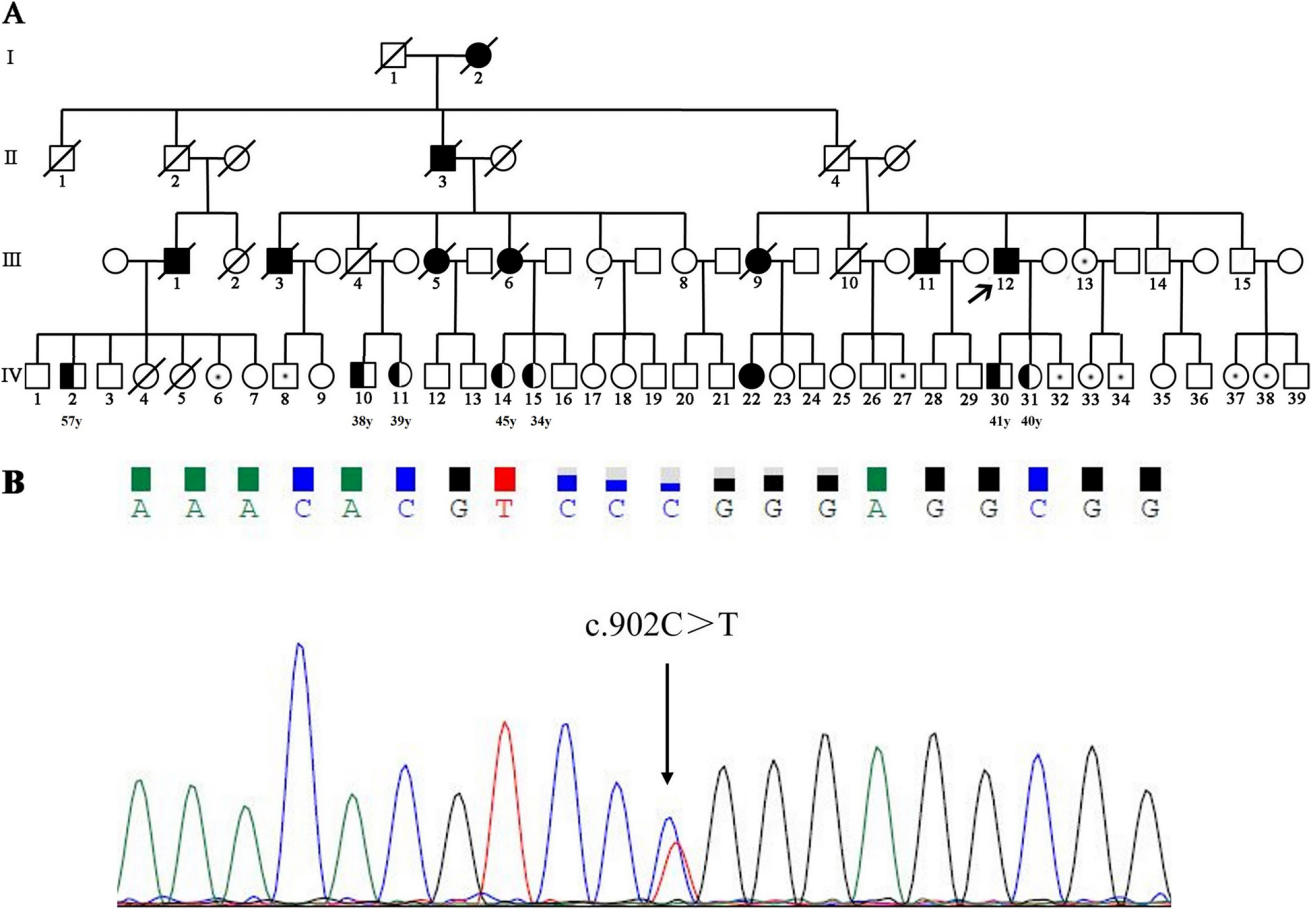
Pedigree and gene mutation of the family. **A** Illustration of pedigrees. Filled symbols, affected subjects; black spots, noncarriers; open symbols, subjects without any clinical symptoms and without genetic testing; diagonal line through a symbol, death; short arrow, proband; half-filled symbols, mutation carriers without any clinical symptoms; circles, females; squares, males. **B** Sanger sequence showing the mutation of MAPT (p.P301L, c.902C>T)

The other participants reported no clinical symptoms, such as declines in language and cognitive function, psychiatric and behavioural symptoms or motor dysfunction, although genetic testing revealed that 7 of them were also MAPT P301L mutation carriers (IV-2, IV-10, IV-11, IV-14, IV-15, IV-30 and IV-31); the inheritance pattern was autosomal dominant transmission. The other 9 participants (III-13, IV-8, IV-32, IV-6, IV-27, IV-33, IV-34, IV-37 and IV-38) did not carry this mutation. The pedigree chart and Sanger sequencing–verified MAPT mutation of the proband are shown in Fig. 1. By consulting PubMed and literature published in Chinese, including the China National Knowledge Infrastructure (CNKI) and Wanfang, we found that a total of 7 patients from 5 families who were diagnosed with svPPA caused by the MAPT P301L mutation had been reported worldwide; in addition, 2 patients were included in the present study (Table 1).

**Table 1:** Published information on patients with svPPA caused by P301L MAPT mutation

Case (family-patient)	AAO (years)	AAD (years)	Family history	Country	Reference
1-1	46	Alive	Yes	Japan	Ishizuka et al. [21]
1-2	53	60	Yes	Japan	Ishizuka et al. [21]
1-3	48	Alive	Yes	Japan	Ishizuka et al. [21]
2-1	46	56	Unknown	Spain	Borrego-Écija et al. [20]
3-1	69	Alive	Unknown	Spain	Borrego-Écija et al. [20]
4-1	43	Alive	Unknown	Spain	Borrego-Écija et al. [20]
5-1	42	56	Yes	USA	Tacik et al. [32]
6-1	62	Alive	Yes	China	Current study
6-2	49	Alive	Yes	China	Current study

*AAO* age at onset, *AAD* age at death

### Cognitive, language and behavioural assessments

Comprehensive cognitive assessments and detailed language evaluations were performed for all participants. The neuropsychological and ABC scores of representative subjects, including two patients (III-12 and IV-22), two mutation carriers (IV-2 and IV-30) and two noncarriers (IV-32 and IV-27), are shown in Tables 2 and 3, respectively. Both patients showed impairment in global cognitive function (III-12: MMSE=17, MoCA=10; IV-22: MMSE=17, MoCA=11). In addition to the naming problems measured with BNT, these two patients showed poor performance on most tests across various cognitive domains, including the TMT-A, TMT-B, SDMT, Stroop, AVLT, BVMT-R, COWAT and AFT. In terms of ABC testing, both patients showed severe deficits in naming and comprehension tasks, in particular, single words compared with sentence comprehension, and had relatively preserved abilities in repetition. Generally, fluent speech was impaired in III-12 and preserved in IV-22. Functional autonomy was still preserved in both patients.

**Table 2:** Neuropsychological assessment in representative participants

	svPPA *N*=2		Presymptomatic carriers *N*=7			Noncarriers *N*=9			*P*-value
Representative subjects	III-12	IV-22	IV-2	IV-30	Mean (SD)	IV-32	IV-33	Mean (SD)	
Age (years)	64	50	57	41	42 (7)	35	36	41 (12)	0.865
Years of education	9	12	5	12	8 (2)	9	12	10 (7)	0.421
MMSE	17	17	23	30	27 (3)	29	30	24 (7)	0.368
MoCA	10	11	24	26	25 (1)	27	27	21 (9)	0.248
TMT part A (seconds)	UC	66	84	30	42 (18)	34	29	42 (12)	0.576
TMT part B (seconds)	UC	180	234	60	96 (60)	36	60	72 (18)	0.357
Stroop words	40	66	60	72	73 (12)	74	95	85 (18)	0.197
Stroop colours	26	25	50	63	61 (15)	57	60	68 (25)	0.51
Stroop interference	14	2	11	32	30 (11)	23	23	33 (15)	0.724
SDMT	8	32	10	50	43 (17)	44	43	59 (13)	0.082
AVLT total learning	16	45	32	43	42 (14)	52	40	54 (11)	0.091
AVLT delayed recall	0	9	10	11	10 (2)	11	11	9 (5)	0.552
AVLT recognition	2	9	5	14	14 (0.7)	11	12	13 (3)	0.321
BVMT-R total learning	7	6	16	22	21 (8)	30	30	21 (11)	0.942
BVMT-R delayed recall	4	2	6	7	9 (3)	12	11	9 (4)	0.906
BVMT-R recognition	5	1	6	5	6 (3)	6	6	5 (2)	0.122
COWAT	3	2	9	17	6 (3)	21	20	6 (3)	0.611
AFT	7	1	10	14	15 (5)	21	20	18 (6)	0.316
BNT	12	6	30	30	29 (3)	29	29	28 (4)	0.639
Digit span forward	10	16	10	13	14 (2)	14	12	12 (4)	0.201
Digit span backward	3	5	5	7	8 (3)	6	5	7 (4)	0.404
ADL	20	20	20	20	20 (0)	20	20	20 (0)	ND
NPI	13	0	0	1	0.9 (0.9)	0	0	0.8 (1.3)	0.893

The mean (SD) values were derived from all 7 presymptomatic carriers and all 9 noncarriers. *P*-value suggests a difference between presymptomatic carriers and noncarriers. *ND* not defined because the two values coincided, *MMSE* Mini-Mental State Examination, *MoCA* Montreal Cognitive Assessment, *TMT* Trail Making Test, *SDMT* Symbol Digit Modalities Test, *AVLT* Auditory Verbal Learning Test, *BVMT-R* Brief Visuospatial Memory Test–Revised, *COWAT* Controlled Oral Word Association Test, *AFT* Animal Fluency Test, *BNT* Boston Naming Test, *ADL* Activities of Daily Living, *NPI* Neuropsychiatric Inventory

**Table 3:** The ABC testing in representative participants

	svPPA *N*=2		Presymptomatic carriers *N*=7			Noncarriers *N*=9			*P*-value
Representative subjects	III-12	IV-22	IV-2	IV-30	Mean (SD)	IV-32	IV-33	Mean (SD)	
*Spontaneous speech*/100									
Information content	0	50	100	100	100 (0)	100	100	100 (0)	ND
Fluency	0	89	100	100	100 (0)	100	100	100 (0)	ND
Repetition	81	93	84	96	96 (6)	100	98	97 (5)	0.869
Responsive naming	50	60	100	100	100 (0)	100	100	97 (10)	0.396
*Comprehension*/100									
Yes/no questions	46	80	93	100	99 (3)	100	100	96 (13)	0.515
Auditory word/picture matching	62	82	94	97	100 (0)	100	100	100 (0)	ND
Following directions	45	79	100	100	100 (0)	100	100	100 (0)	ND
*Reading*/100									
Oral reading	80	80	100	100	100 (0)	100	100	100 (0)	ND
Auditory word and print matching	80	90	100	100	100 (0)	100	100	100 (0)	ND
Reading and following written directions	26	67	100	100	100 (0)	100	100	98 (5)	0.262
Reading and filling in the blanks	13	47	100	100	100 (0)	100	100	100 (0)	ND
*Writing*/100									
Spontaneous writing	0	100	100	100	97 (8)	100	100	97 (8)	ND
Writing name and address	100	100	100	100	100 (0)	100	100	100 (0)	ND
Written naming of pictures	20	70	100	100	100 (0)	100	100	100 (0)	ND
Writing from dictation	37.5	58	100	100	100 (0)	100	100	100 (0)	ND
Copying	100	100	100	100	100 (0)	100	100	100 (0)	ND

The mean (SD) values were derived from all 7 presymptomatic carriers and all 9 noncarriers. *ND* not defined because the two values coincided, *ABC* Aphasia Battery in Chinese

As a mutation carrier, IV-2 also had slightly reduced scores on the MMSE and MoCA and impairments in processing speed and executive function (TMT-A and B, SDMT and Stroop), episodic memory (learning, delayed recall and recognition components of the AVLT), verbal fluency (COWAT and AFT) and repetition and comprehension on the ABC test relative to Chinese norms, although he did not display any clinical manifestations. For the mutation noncarriers, IV-6 and III-13 also exhibited poor performance in cognitive testing, which was considered to be attributable to cerebrovascular disease according to the MRI scan of subject IV-6 and illiteracy and potential neurological or mental disorders in subject III-13. All neuropsychological and language testing scores in other participants, including both mutation carriers and noncarriers, were mostly in the normal range.

### Structural MRI reading

Eight carriers and eight noncarriers completed multimodal MRI scans. Structural MRI images showed bilateral but left-predominant atrophy of the anterior temporal lobes and bilateral frontal lobe atrophy in subject IV-22. Cerebrovascular lesions, including multiple lacunes (5 areas) and severe WM hyperintensities, were observed in subject IV-6 and were more obvious than age-related changes. No clinically significant abnormalities were observed in the other participants according to the visual readings.

### PET scan reading

**Fig. 2:**
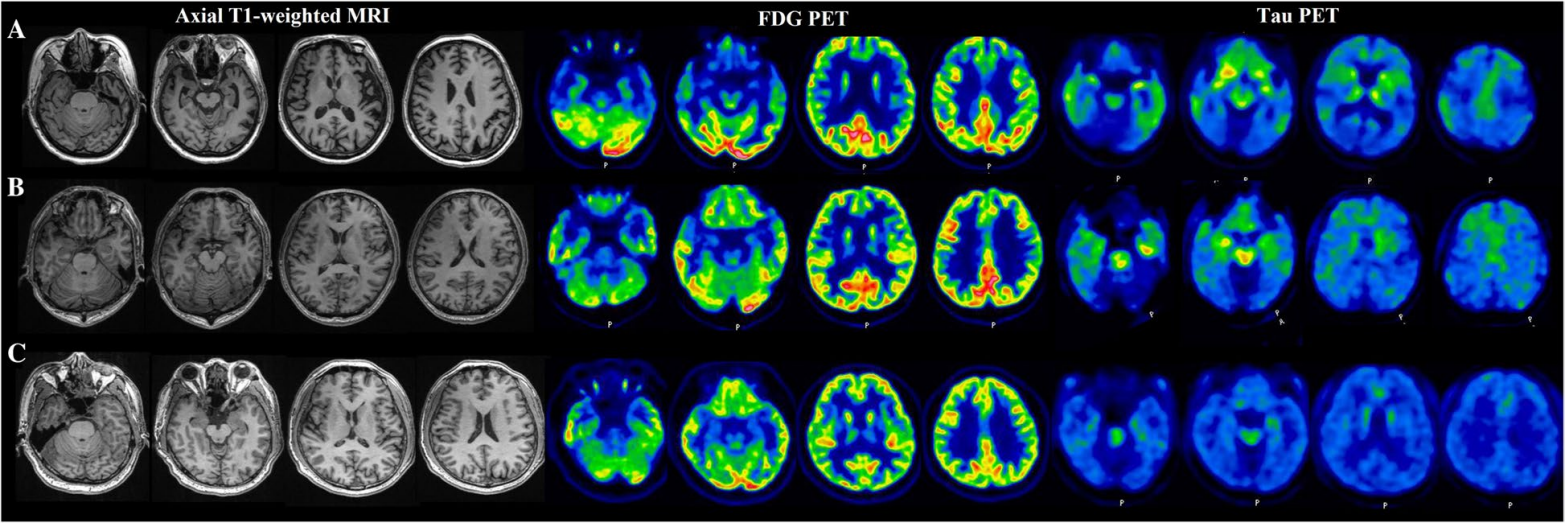
Neuroimaging presentations of the svPPA patient and two presymptomatic carriers. **A** IV-22 (svPPA patient): bilateral frontal atrophy and bilateral but left-dominant atrophy of the anterior temporal lobes on brain MRI; hypometabolism in the bilateral temporal cortex, frontal cortex, insula and caudate nucleus on FDG PET; and diffuse tracer binding in the temporal and frontal lobes of the cerebrum on tau-PET. **B** IV-2 (presymptomatic carrier with abnormal cognitive assessment): no visually obvious atrophy of the bilateral frontal and temporal lobes on brain MRI; focal hypometabolism of the bilateral frontal lobes on FDG PET; diffuse tracer binding in the temporal and frontal lobes of the cerebrum on tau-PET. **C** IV-30 (presymptomatic carrier with normal cognitive assessment): no obvious atrophy, hypometabolism or tau accumulation on brain MRI and PET scans. There is “off-target” binding in brain areas without tau deposition, such as the brain stem and the basal ganglia

Three mutation carriers completed PET scans. The FDG PET scans of patient IV-22 exhibited hypometabolism located predominantly in the bilateral temporal cortices and frontal cortices and mildly in the insula and caudate nucleus. The tau PET scans showed diffuse tracer binding in the temporal and frontal cortices, which largely overlapped with the hypometabolic areas found in the FDG PET scans. For IV-2, who had no symptoms but abnormal cognitive testing, focal hypometabolism in the bilateral frontal lobes and diffuse tracer binding in the bilateral temporal and frontal lobes were found in the FDG and tau PET scans, respectively. IV-30, who exhibited no symptoms and had normal cognition, showed normal metabolism on the FDG PET scans and negative results on the tau PET scans. Structural MRI, FDG and tau PET images of these three participants are shown in Fig. 2.

### Concentrations of plasma biomarkers

**Fig. 3:**
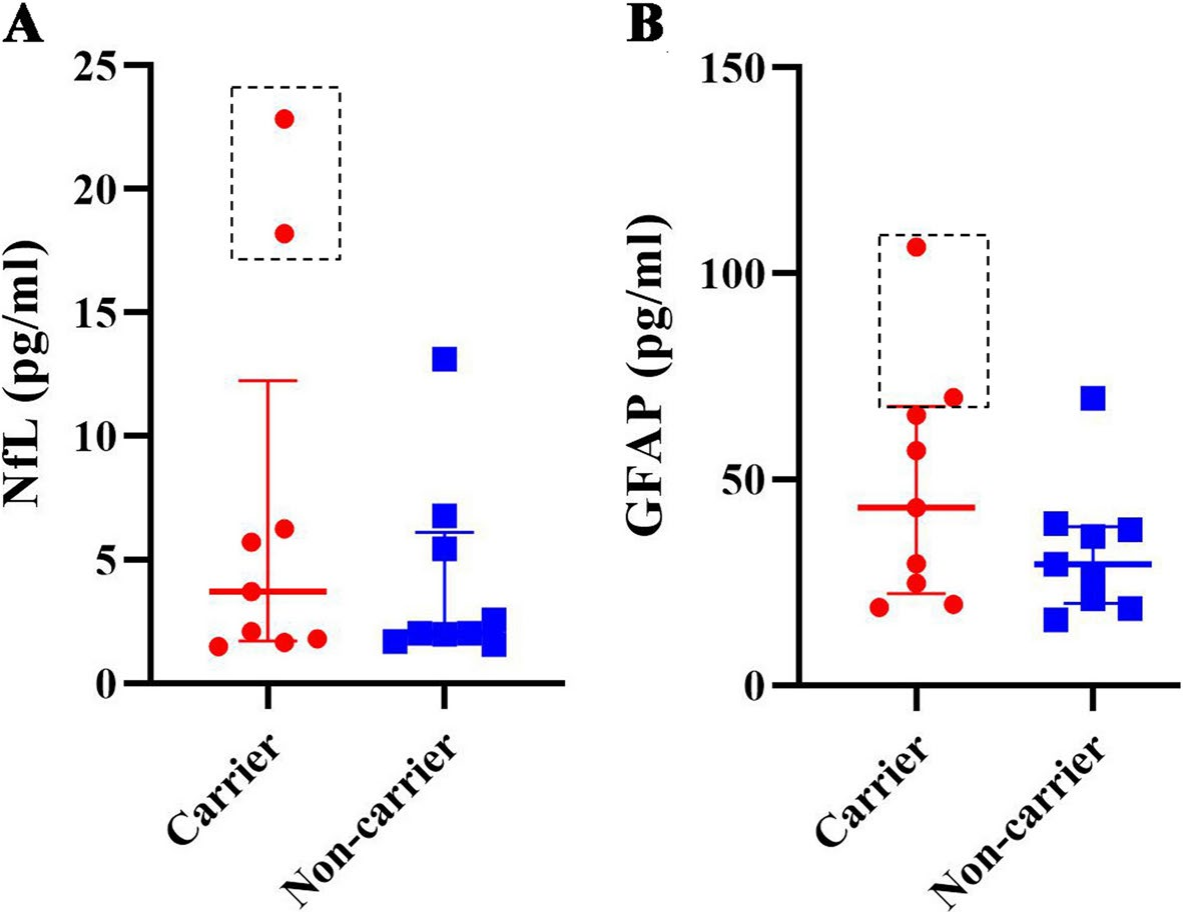
Plasma NfL and GFAP concentrations in mutation carriers and noncarriers. There was no difference in **A** NfL or **B** GFAP between 9 mutation carriers and 9 noncarriers, although the levels of NfL and GFAP were higher in the two patients designated III-12 and IV-22 (indicated by the dashed boxes) than in presymptomatic carriers or noncarriers

As shown in Fig. 3, after adjusting for age, there were no differences between 9 mutation carriers and 9 noncarriers in either NfL (median=3.72 pg/ml [interquartile range (IQR): 1.48–22.85] vs. median=2.03 pg/ml [IQR: 1.56–13.11], respectively, *P*>0.05) or GFAP (median=43.18 pg/ml [IQR: 18.98–106.44] vs. median=29.42 pg/ml [IQR: 15.97–69.69], *P*>0.05). However, the NfL and GFAP levels were higher in two patients (III-12: NfL=18.2 pg/ml, GFAP=106.44 pg/ml; IV-22: NfL=22.85 pg/ml, GFAP=69.77 pg/ml) than in both the presymptomatic carriers and noncarriers. The plasma NfL and GFAP concentrations were significantly correlated with each other in the mutation carriers (*r*=0.774, *P*<0.05) but not in the noncarriers (*r*=0.188, *P*>0.05).

### Comparison of CBF between mutation carriers and noncarriers

**Fig. 4:**
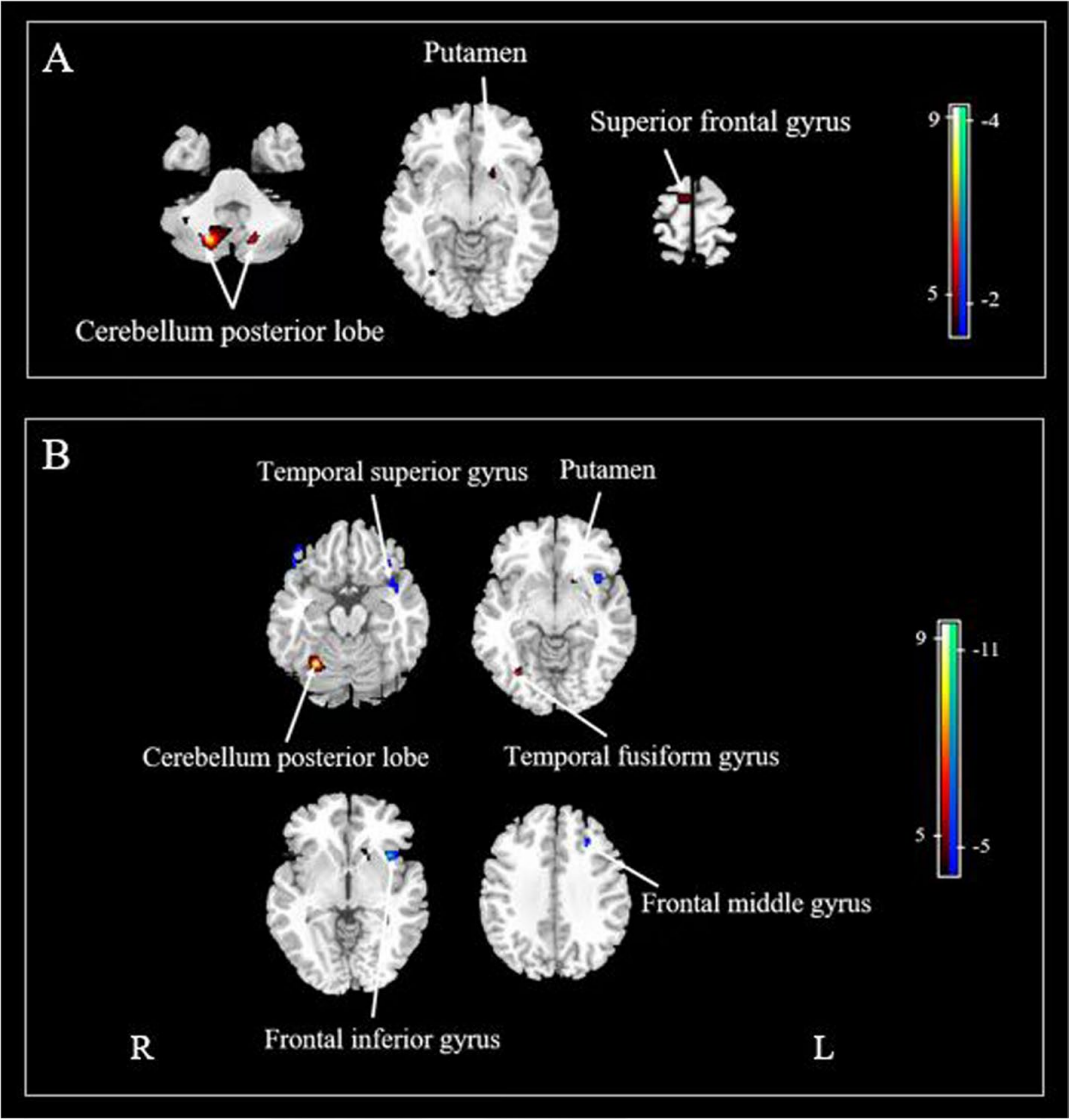
Regional CBF differences between mutation carriers and noncarriers. **A** Without adjusting for the global values, increased regional CBF was observed in mutation carriers. **B** After ANCOVA normalization to the global values, regions of relatively decreased and relatively increased regional CBF were both observed in the mutation carriers. A voxel-level peak threshold of *P* < 0.001 was used to overlay SPM maps onto a standard MRI brain template. Warm colours indicate regions with increased CBF in mutation carriers compared with noncarriers, while cold colours indicate regions with decreased CBF

For the CBF analyses, we further excluded two noncarriers, subjects IV-6 and III-13, who showed poor performance in cognitive testing to avoid any influences due to other neurological diseases. Without adjusting for the global value, mutation carriers showed increased absolute CBF in the right superior frontal gyrus, bilateral cerebellum posterior lobes, and left putamen, but there were no decreases in absolute CBF levels in any brain areas relative to the noncarriers (Fig. 4A and Table 4).

**Table 4:** Regions showing increased absolute CBF without normalization

Structure	BA	X	Y	Z	T	Volume (ml)
Right superior frontal gyrus	6	5	−8	74	5.00	0.83
Right posterior lobe of the cerebellum		18	−72	−44	9.2	12.68
Left posterior lobe of the cerebellum		−17	−71	−39	5.83	0.618
Left putamen		−20	12	−8	4.94	0.44

*BA* Brodmann area

After ANCOVA normalization for the global values, both relative decreases and relative increases in regional CBF were observed in the mutation carriers (Fig. 4B and Table 5). Specifically, CBF in the bilateral frontal inferior gyri, left frontal middle gyrus and left temporal superior gyrus was reduced in carriers compared to noncarriers. The regions with relatively increased CBF in the carriers were mainly distributed in the right temporal fusiform gyrus, right cerebellum posterior lobe and left putamen.

**Table 5:** Regions showing relative CBF changes after adjustment for global values (ANCOVA normalization)

Structure	BA	X	Y	Z	T	Volume (ml)
Relative decrease in CBF						
Left frontal inferior gyrus	47	−38	17	−3	11.75	2.52
Right frontal inferior gyrus	47	48	29	−17	6.23	0.50
Left frontal middle gyrus	9	−30	27	36	5.99	0.378
Left temporal superior gyrus	38	−44	6	−21	5.63	2.52
Relative increase in CBF						
Right temporal fusiform gyrus		32	−69	−11	6.22	2.74
Right posterior lobe of the cerebellum	19	27	−60	−18	9.29	2.74
Left putamen		−18	14	−8	5.83	0.36

*BA* Brodmann area

**Fig. 5:**
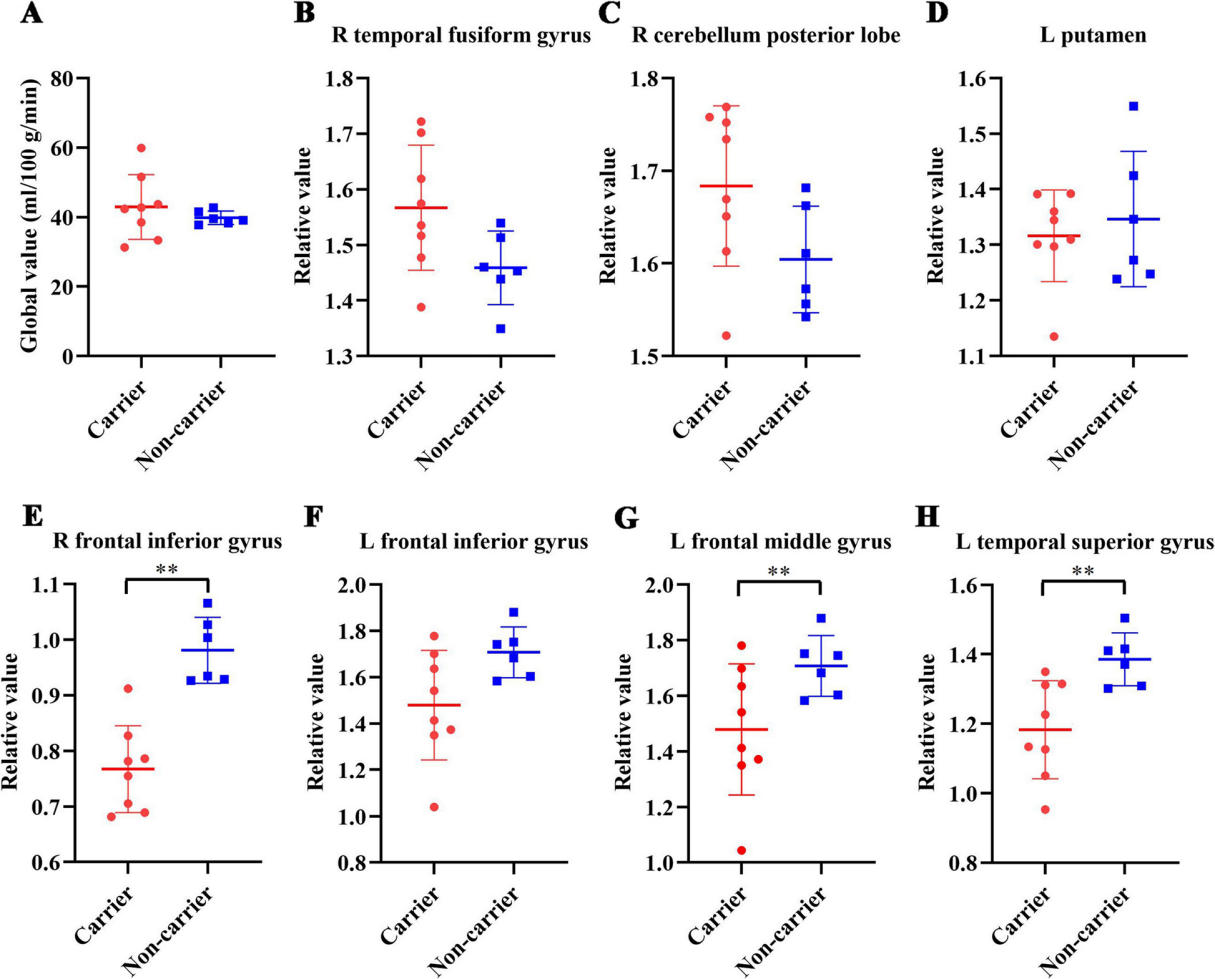
Differences in global and relative CBF values for representative regions between mutation carriers and noncarriers. **A** Comparisons of global values. **B**–**H** Comparisons of relative values in the right temporal fusiform gyrus (32, −69, −11), right cerebellar posterior lobe (27, −60, −18), left putamen (−18, 14, −8), right frontal inferior gyrus (48, 29, −17), left frontal inferior gyrus (−38, 17, −3), left frontal middle gyrus (−30, 27, 36) and left temporal superior gyrus (−44, 6, −21). **P<0.01

There was no significant difference in global CBF between the mutation carriers (42.93±3.30 ml/100 g/min) and noncarriers (39.83±0.79 ml/100 g/min). The results did not change after adjustment for age. Sample plots for the global CBF and relative values in representative regions, including the right cerebellar posterior lobe, right temporal fusiform gyrus, left putamen, left temporal superior gyrus, left frontal inferior gyrus, right frontal inferior gyrus and left frontal middle gyrus, are shown in Fig. 5.

## Discussion

In the present study, we described a Chinese family with svPPA that was caused by the MAPT P301L mutation. All subjects in this family, including living and deceased subjects, showed semantic memory impairment as the first and most prominent symptom. In addition to language disabilities in naming and comprehension, global cognition and various specific cognitive domains were impaired in the svPPA patients, even in the early or preclinical stages. The patients included in this study presented typical imaging features of svPPA, such as atrophy, hypometabolism and tau deposition in the bilateral temporal and frontal cortices according to the structural MRI, FDG PET and tau PET scans, respectively. Although the plasma NfL and GFAP concentrations were elevated in the two diagnosed patients, there were no differences in the levels of these biomarkers between carriers in general and noncarriers in general. However, absolute increases in regional CBF and relative decreases in regional CBF measured with ASL were observed in the mutation carriers. To our knowledge, this is the first svPPA family that is associated with a MAPT P301L mutation to be reported in a Chinese population. This study provides an ideal model for inferring the underlying pathology and pathophysiological processes in svPPA due to tauopathies.

svPPA is exceptional among FTLD phenotypes in the sense that it is typically sporadic, with the least likelihood of a genetic cause [7]; only 2–7% of patients with svPPA have a family history [33]. Genetic screening of the common genes associated with AD and FTLD in a large cohort of patients with PPA showed that causative and risk-associated variants were rarely present, particularly for the svPPA and lvPPA phenotypes [34]. The mutations that are most commonly reported in svPPA patients who have a highly positive family history include mutations in transactive response DNA-binding protein (TARDBP), GRN and C9orf72 [35–37], which all cause the main pathology of FTLD-TDP [7]. However, tauopathies caused by mutations in the MAPT gene are frequently associated with a clinical phenotype of bvFTD or parkinsonism, although some patients may develop semantic impairment as the disease progresses [19, 33, 38].

In the present study, we found the MAPT P301L mutation in patients with svPPA from a large family across four generations. A few patients with the MAPT P301L mutation had previously been reported in China; these patients mostly presented with bvFTD [17–19], as well as declines in language and calculation abilities [39]. One study from Spain showed that behavioural changes constituted the most common initial symptoms, followed by language impairment, cognitive dysfunction and possible parkinsonism [20]. Interestingly, three patients from that family were clinically diagnosed with bvFTD, svPPA and AD, respectively, which indicated potential heterogeneity in the clinical manifestations of the P301L mutation [20]. Another study also reported phenotypic heterogeneity in a Caucasian family: one patient was diagnosed with svPPA accompanied by refractory seizures, and both his sibling and cousin were diagnosed with bvFTD [32]. Overall, while the P301L mutation can lead to diverse clinical phenotypes, svPPA is rarely reported. Previously, the only such family reported anywhere in the world was a family in Japan; five people in two generations showed semantic memory impairment, with loss of word meanings as the first and most prominent symptom, and were diagnosed with svPPA with the P301L mutation [21].

To date, this is the largest reported svPPA family with the MAPT P301L mutation worldwide. In this study, the age at disease onset (range 48–60 years) and disease duration (6–7 years) of individuals in the pedigree were consistent with those of previously reported patients [20]. All patients uniformly presented marked difficulty in naming and semantic memory impairment at an early stage with dementia onset. Apart from language dysfunction, particularly in naming and comprehension, we observed impairments in global cognition and various cognitive domains, such as processing speed and executive function, verbal and spatial episodic memory, and verbal fluency, in the two svPPA patients and even in a presymptomatic mutation carrier. In support of this finding, previous studies also reported a decline in MMSE scores [40], impairments in verbal memory and executive function [41] and other language problems, such as verbal fluency, repetition and spontaneous speech [40, 41], in svPPA. For presymptomatic MAPT mutation carriers, naming difficulty was still the most frequent observation from neuropsychological testing based on a large longitudinal cohort in North America (ARTFL/LEFFTDS) [42], and cognitive dysfunction in various domains, such as episodic memory [43] and phonemic verbal fluency [44], was also found in previous studies.

Consistent with previous findings [4, 45, 46], brain atrophy, hypometabolism and tau deposition were primarily distributed in the anterior temporal and frontal lobes with a left-predominant asymmetry in the svPPA patient in the present study, which indicated an overlap between tau pathology and neurodegeneration. For the presymptomatic mutation carrier with objective cognitive impairment (subject IV-2), the distribution of tau deposition was more extensive than the region with hypometabolism. This finding was supported by a previous study showing that tracing tau protein with PET was more sensitive in detecting neuropathological changes than structural MRI and single-photon emission computed tomography (SPECT) in patients with svPPA [47]. However, a young presymptomatic mutation carrier who exhibited no decline upon cognitive and language testing (subject IV-30, aged 41 years) showed negative results in both the tau and FDG PET scans, which suggests that these imaging modalities have limited utility in screening people at risk for MAPT mutation.

We found that the plasma NfL and GFAP levels, which have been recognized as biomarkers for neurodegeneration and neuroinflammation, respectively, were not different between mutation carriers and noncarriers, although both biomarkers were higher in the two patients than in family members without symptoms. CSF and blood NfL levels, which were shown to be strongly correlated [48], were suggested to be candidate markers of disease onset, prognosis and disease progression in FTLD. Similar to our findings, previous studies also showed that the blood NfL levels were higher in svPPA patients than in healthy controls [49] and in FTLD patients than in either presymptomatic carriers or healthy controls but did not differ between the latter two groups [50]. In addition, recent studies reported that serum GFAP levels were significantly higher in patients with FTLD than in healthy controls and were correlated with cognitive dysfunction and disease severity [51]. Our results supported the notion that both blood NfL and GFAP are potential predictors for disease onset in patients with svPPA but might not be useful in identifying people at risk. We also found that the plasma NfL and GFAP concentrations were significantly correlated with each other in mutation carriers but not in noncarriers. However, this correlation was observed not only in presymptomatic and symptomatic carriers of mutations (including MAPT mutations) but also in noncarriers in a large cohort from the Genetic Frontotemporal Dementia Initiative (GENFI) study [52].

CBF changes have been recognized as an early pathophysiological process in neurodegenerative diseases even before their core neuropathology, as observed in AD [53]. In the present study, absolute increases but no decreases in regional CBF were observed in the frontal regions, cerebellum and deep GM in mutation carriers compared to noncarriers. A previous study [54] also reported hyperperfusion in some frontal and temporoparietal areas measured with ASL in addition to temporal hypoperfusion in svPPA patients, indicating a compensatory mechanism of perfusion and/or an alteration in the functional brain network. It was suggested that CBF changes began to occur at ~12.5 years before their expected age of symptom onset and included a period of slight increase in all mutation (including C9orf72, GRN and MAPT) carriers who were included in the GENFI study [43], although no obvious CBF changes were reported in MAPT mutation presymptomatic carriers in either the GENFI study or another ASL study [55]. Moreover, after global normalization, we observed relative decreases in CBF mainly in the frontal and temporal cortices in mutation carriers compared to noncarriers, which explained the early impairments in comprehension, episodic memory and executive function. Interestingly, dysfunction in CBF regulation was recently observed in mice expressing MAPT P301S or P301L mutations, even before tauopathy and neurodegeneration [56, 57]. Taken together, ASL MRI is a promising measure for early identification and is worthy of further investigation in preclinical svPPA patients with tauopathy.

The mutation carriers identified in our present study were 34–57 years old, which may be too young for disease onset. The GENFI study indicated that structural imaging and cognitive changes could be identified 5–15 years before the expected onset of symptoms in presymptomatic MAPT carriers [58]. In the present study, individual IV-2, who showed imaging and cognitive changes upon testing but did not exhibit clinical symptoms, is 57 years of age, which is 5 years earlier than the onset age of the proband (III-12). Long-term follow-up of mutation carriers will help to establish a dynamic biomarker model for MAPT- or tauopathy-related FTLD, including CBF changes, tau accumulation, neuroinflammation, neurodegeneration and cognitive and linguistic impairments.

## Conclusions

Although rare, tau pathology attributed to the MAPT mutation could lead to FTLD with an svPPA phenotype. Comprehensive neuropsychological assessments, FDG and tau PET scans, and plasma NfL and GFAP levels are useful and more sensitive than evaluating symptoms and visual reading of brain atrophy on MRI in the early identification of genetic svPPA patients with the MAPT mutation. Moreover, CBF measured with ASL is a promising measure to detect early neurobiological changes, even before neurodegeneration or tau measurements.

## Data Availability

The datasets generated and analysed during the current study are available from the corresponding author upon reasonable request.

## Abbreviations

FTLD: Frontotemporal lobar degeneration
AD: Alzheimer’s disease
bvFTD: Behavioural variant frontotemporal dementia
PPA: Primary progressive aphasia
nfvPPA: Nonfluent/agrammatic variant PPA
svPPA: Semantic variant PPA
TDP: TAR DNA-binding protein
FUS: Fused in sarcoma
C9orf72: Chromosome 9 open reading frame 72
GRN: Progranulin
MAPT: Microtubule-associated protein tau
FTDP-17: Frontotemporal dementia and parkinsonism linked to chromosome 17
CBF: Cerebral blood flow
ASL: Arterial spin labelling
NGS: Next-generation sequencing
Aβ: Amyloid-β
GM: Grey matter
NfL: Neurofilament light chain
GFAP: Glial fibrillary acidic protein
WM: White matter
FWHM: Full width at half maximum
VOI: Volume of interest
TARDBP: Transactive response DNA-binding protein
